# MLL-AF4 binds directly to a BCL-2 specific enhancer and modulates H3K27 acetylation

**DOI:** 10.1016/j.exphem.2016.11.003

**Published:** 2017-03

**Authors:** Laura Godfrey, Jon Kerry, Ross Thorne, Emmanouela Repapi, James O.J. Davies, Marta Tapia, Erica Ballabio, Jim R. Hughes, Huimin Geng, Marina Konopleva, Thomas A. Milne

**Affiliations:** a Weatherall Institute of Molecular Medicine, MRC Molecular Haematology Unit, University of Oxford, Headington, Oxford, UK; b Weatherall Institute of Molecular Medicine, Computational Biology Research Group, University of Oxford, Headington, Oxford, UK; c Department of Laboratory Medicine, University of California San Francisco, San Francisco, CA; d Department of Leukemia, University of Texas MD Anderson Cancer Center, Houston, TX

## Abstract

Survival rates for children and adults carrying mutations in the *Mixed Lineage Leukemia* (*MLL*) gene continue to have a very poor prognosis. The most common *MLL* mutation in acute lymphoblastic leukemia is the t(4;11)(q21;q23) chromosome translocation that fuses *MLL* in-frame with the *AF4* gene producing MLL-AF4 and AF4-MLL fusion proteins. Previously, we found that MLL-AF4 binds to the *BCL-2* gene and directly activates it through DOT1L recruitment and increased H3K79me2/3 levels. In the study described here, we performed a detailed analysis of MLL-AF4 regulation of the entire BCL-2 family. By measuring nascent RNA production in MLL-AF4 knockdowns, we found that of all the BCL-2 family genes, MLL-AF4 directly controls the active transcription of both *BCL-2* and *MCL-1* and also represses *BIM* via binding of the polycomb group repressor 1 (PRC1) complex component CBX8. We further analyzed MLL-AF4 activation of the *BCL-2* gene using Capture-C and identified a *BCL-2*-specific enhancer, consisting of two clusters of H3K27Ac at the 3′ end of the gene. Loss of MLL-AF4 activity results in a reduction of H3K79me3 levels in the gene body and H3K27Ac levels at the 3′ *BCL-2* enhancer, revealing a novel regulatory link between these two histone marks and MLL-AF4-mediated activation of *BCL-2.*

## Highlights

• Mixed lineage leukemia (MLL)-AF4 directly controls the active transcription of both *BCL-2* and *MCL-1.*
• MLL-AF4 represses *BIM* via recruitment of the polycomb group repressor 1 member CBX8.
• Capture-C and chromatin immunoprecipitation sequencing are used to identify a *BCL-2*-specific enhancer.
• The *BCL-2* enhancer consists of two clusters of H3K27Ac at the 3′ end of the gene.
• MLL-AF4 controls H3K79me3 in the *BCL-2* gene body and H3K27Ac at the enhancer.

Survival rates for children diagnosed with acute lymphoblastic leukemia (ALL) have drastically improved and, in some cases, are now approaching 90% 1, 2. However, even with this progress, adults with ALL still have a very poor prognosis [3], and childhood ALL cases that relapse often exhibit drug resistance and remain difficult to treat [4]. In addition, specific subgroups of ALL patients, especially those carrying rearrangements of the *mixed lineage leukemia* (*MLL*) gene 5, 6, still have very poor survival outcomes [5]. Even with the advent of novel therapeutic approaches such as CAR T-cell therapy, *MLL* ALLs often rapidly relapse as acute myeloid leukemia (AML) [7], and thus, treatment of *MLL* ALL patients remains an unmet need.

The most common *MLL* rearrangement (MLLr) in ALL is the t(4;11)(q21;q23) chromosome translocation that fuses *MLL* in-frame with the *AF4* gene, producing MLL-AF4 and AF4-MLL fusion proteins [8]. The role of AF4-MLL in leukemogenesis is controversial, as AF4-MLL has transformation capabilities [9], but is expressed in only 50% to 80% of patients 10, 11 and thus may not be necessary for leukemic maintenance. MLL-AF4, however, is essential for leukemic maintenance [12] and this, combined with the fact that t(4;11) leukemias have very few cooperating mutations [10], suggests that a detailed understanding of the function of MLL-AF4 may aid in the design of targeted therapies.

The MLL-AF4 fusion protein binds to gene targets and is proposed to cause inappropriate gene activation through multiple transcription elongation and epigenetic mechanisms 13, 14, 15, 16. We recently reported that MLL-AF4 activates important gene targets such as *RUNX1*, *HOXA9*, and *BCL-2* 17, 18, mainly through recruitment of the disruptor of telomeric silencing 1-like (DOT1L) protein (the only known histone 3 lysine 79 methylation methyltransferase [19]) and increased H3K79me2/3 levels [18]. Past work has suggested that MLL-AF4 leukemias are particularly sensitive to loss of BCL-2 activity [20], and we were able to extend this observation by illustrating that BCL-2 is a key therapeutic target in MLL-AF4 leukemias [18].

BCL-2 is part of a family of proteins that controls apoptosis, and perturbed expression of BCL-2 family members is associated with tumorigenesis and resistance to cancer treatments 21, 22, 23. The BCL-2 family is divided into antiapoptotic factors (e.g., BCL-2, MCL-1, BCL-XL, BCL-w, BCL2-A1), pore-forming proapoptotic factors (BAX and BAK), and BH3-only proteins (e.g., BIM, BID, and BAD). Recent work suggests that the primary role of the BH3-only proteins is to sequester the antiapoptotic factors (e.g., BCL-2), allowing the initiation of mitochondrial outer membrane permeabilization (MOMP), the first irreversible step of apoptosis [24]. Therefore, maintaining normal cellular homeostasis requires maintenance of a precise balance between antiapoptotic and BH3-only protein levels for the control of cell survival. The MLL-AF4 protein disrupts this balance by directly binding to the *BCL-2* gene 17, 18, 25, 26, causing increased gene expression and elevated BCL-2 protein levels [18].

ABT-199 (also known as venetoclax) is a highly specific BH3 mimetic that can preferentially target BCL-2 activity [27]. In ALL, MLL-AF4 leukemias are distinctly sensitive to treatment with venetoclax alone 18, 28. However, even among MLLr samples, a subset either is resistant to venetoclax [28] or relapses after an early initial response [18]. Resistance to BH3 mimetic monotherapy usually occurs via upregulation of alternative antiapoptotic factors such as MCL-1 and BCL-XL 29, 30, 31, 32, 33. Notably, high-level *MCL-1* gene expression is also known to be associated with resistance to treatment in MLLr samples [34]. We found that the venetoclax treatment strongly synergized with standard chemotherapeutic agents, likely in part because of the treatment-induced reduction of MCL-1 and BCL-XL protein levels [18]. More interestingly, we also found that venetoclax treatment synergized with DOT1L inhibitors (also confirmed in a more recent study [35]), although it is unclear if the mechanism of synergy is through downregulation of other BCL-2 family proteins. In our past work, other *BCL-2* family genes such as *MCL-1*, *BIM*, *BAX*, and *BCL-XL* did not appear to be regulated by MLL-AF4 or DOT1L [18], but admittedly this could have been due to the sensitivity of the assays used for the analysis.

To more carefully analyze MLL-AF4- and DOT1L-mediated regulation of *BCL-2* family genes, here we use nascent RNA sequencing (RNA-seq) coupled with MLL-AF4 siRNA knockdowns and DOT1L inhibitor treatments to illustrate that MLL-AF4 directly activates both *BCL-2* and *MCL-1*. Interestingly, we also find that MLL-AF4 represses the *BIM* gene through the polycomb group repressor 1 (PRC1) component CBX8, although this has little overall effect on BIM protein levels. We further analyze MLL-AF4 activation of the *BCL-2* gene using Capture-C and identify a *BCL-2* enhancer. Loss of MLL-AF4 results in a concurrent reduction of H3K27Ac at the enhancer as well as H3K79me3 in the gene body, revealing a novel interplay between these two histone marks in MLL-AF4-mediated activation of *BCL-2*.

## Methods

### Cell lines and cultures

SEM cells [36] were purchased from DSMZ (www.cell-lines.de) and cultured in Iscove's modified Dulbecco's medium (IMDM) supplemented with 10% fetal calf serum. For treatment studies, SEM cells were treated with the DOT1L inhibitor EPZ5676 (Epizyme) for 7 days, with medium and inhibitor changes on days 0, 3, and 6. SEM cells were seeded at a concentration of 0.3 × 10^6^ cell/mL on days 0 and 3 and at a concentration of 0.7 × 10^6^ cell/mL on day 6 prior to harvesting on day 7.

### RNAi assays

MLL-AF4 siRNA was performed as described [18], and sequences were obtained from Thomas et al. [12].

### Reverse transcription polymerase chain reaction

Reverse transcription polymerase chain reaction (RT-PCR) SYBR green primers and Taqman primer/probe sets are as previously described 17, 18 except for the following SYBR green primer sets used in this study:

β2M forward: TGCTGTCTCCATGTTTGATGTATCT
β2M reverse: TCTCTGCTCCCCACCTCTAAGT
YWHAZ forward: ACTTTTGGTACATTGTGGCTTCAA
YWHAZ reverse: CCGCCAGGACAAACCAGTAT
BCL-2 (no2) forward: GTGGATGACTGAGTACCTGAAC
BCL-2 (no2) reverse: GAGACAGCCAGGAGAAATCAA
MCL1 (no2) forward: TGCTTCGGAAACTGGACATC
MCL1 (no2) reverse: GTTACGCCGTCGCTGAAA
MCL1 (no3) forward: AAACGGGACTGGCTAGTTAAA
MCL1 (no3) reverse: CATTCCTGATGCCACCTTCTA
MCL1 (no4) forward: GCATCGAACCATTAGCAGAAAG
MCL1 (no4) reverse: CTCTACATGGAAGAACTCCACAA

### Patient microarray gene expression data

We analyzed a microarray gene expression data set from a St. Jude ALL cohort [37] (*n* = 154) that includes these patient subsets: 18 BCR-ABL1, 8 E2A-PBX1, 15 MLLr, 24 TEL-AML1, 26 hyperdiploid, 11 CRLF2, 11 ERG, and 7 other.

### Western blot analysis

Western blot analysis was performed as previously described [18].

### Chromatin immunoprecipitation assays and -seq

Chromatin immunoprecipitation (ChIP) and ChIP-seq experiments were performed as previously described 17, 18, 38, and BCL family and control gene primer sequences are as previously described [18].

### Antibodies

All antibodies used for Western blot, ChIP and ChIP-seq are as previously described [18] except for P300 (Bethyl, A300-358A, lot 3), E2A (Cell Signaling 12258, lot 1), PBX1/2/3 (Santa Cruz, sc888 lot J0212), cleaved PARP (Cell Signaling 5625), and β-TrCP (Cell Signaling 11984).

### Nascent RNA-seq

Cells (10^8^) were treated with 500 μmol/L 4-thiouridine (4-SU) for 1 hour. Cells were lysed using trizol, and RNA was precipitated with ethanol. 4-SU-Incorporated RNA was biotinylated by labeling with 1 mg/mL Biotin-HPDP for 90 min at room temperature. Following chloroform extraction, labeled RNA was separated using magnetic streptavidin beads. Beads were washed using a magnetic μMACS stand before RNA was eluted in two rounds of elution with 100 μL 100 mmol/L dithiothreitol. RNA was purified using a Qiagen RNeasy MinElute kit. Samples were sequenced on a NextSeq 500 using a high 75 v2 sequencing kit. Nascent RNA-seq experiments were carried out in triplicate.

### Next-Generation Capture-C

Next-generation Capture-C was performed as previously described [39]. A biotinylated DNA oligonucleotide was designed for the promoter of the *BCL2* gene (Integrated DNA Technologies) with the following sequence (5′ to 3′):

Biotin-GATCTCAAGAGCTCGAGAAAAAAAAAAGGCAGCGGCGGCGGCAGATGAATT
ACAATTTTCAGTCCGGTATTCGCAGAAGTCCTGTGATGTTTTCCCCTTCTCGGCAATTTACACGCGCGC

SEM cells (2 × 10^7^) were used in the assay to generate a standard 3C library. Very briefly, the cells were fixed with 2% formaldehyde for 10 min coupled with *Dpn*II digestion and sonication of fragments to 200 bp (see Davies et al. [39] for more details). The biotinylated probe was hybridized to the 3C library (see Davies et al. [39]) for 72 hours, and streptavidin beads (Invitrogen) were used to enrich for fragments hybridized to the BCL-2 promoter oligo. A double-capture step approach was taken, also described in Davies et al. [39]. DNA libraries were prepared using the NEBNext DNA library kit according to the manufacturer's instructions (New England BioLabs, E6040, E7335, and E7500). Libraries were sequenced using the Illumina MiSeq platform with 150-bp paired-end reads. Capture-C analysis was performed using an in-house pipeline to analyze purified BCL-2 promoter fragments for the presence of additional interacting sequences.

### Gene expression analysis

Following quality control (QC) analysis with the fastQC package (http://www.bioinformatics.babraham.ac.uk/projects/fastqc), reads were aligned using STAR [40] against the human genome assembly (NCBI build36 [hg18] UCSC transcripts). Reads that were identified as PCR duplicates using Samtools [41] were discarded. Gene expression levels were quantified as read counts using the featureCounts function [42] from the Subread package [42] with default parameters. The read counts were used for the identification of global differential gene expression between specified populations using the edgeR package [43]. RPKM values were also generated using the edgeR package. Genes were considered differentially expressed between populations if they had an adjusted *p* value (FDR) < 0.05.

### NanoString

NanoString experiments (NanoString Technologies) were performed using nascent RNA samples and a custom-designed array according to the recommendations of the manufacturer. The data were analyzed using NanoStriDE software [44].

### Gene Expression Omnibus accession numbers for ChIP-seq and RNA-seq data

The Gene Expression Omnibus (GEO) accession number for the data discussed in this article is GSE85988 (http://www.ncbi.nlm.nih.gov/geo/query/acc.cgi?acc=GSE85988).

## Results

### MLL-AF4 activates transcription of *BCL-2* and *MCL1*

**Figure 1:**
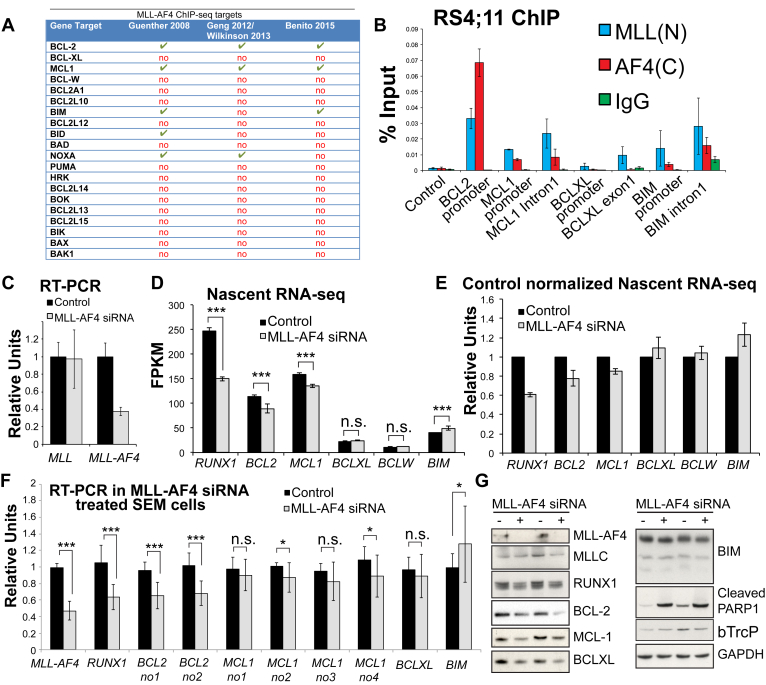
MLL-AF4 maintains *BCL-2* and *MCL-1* transcription and represses *BIM*. (**A**) A table summarizing ChIP-seq data on MLL-AF4 binding to different BCL-2 family target genes in three different data sets in SEM cells 18, 25 or RS4;11 cells 17, 26. (**B**) ChIP in RS4;11 cells using MLL(N), AF4(C), and control (IgG) antibodies confirms MLL-AF4 binding at *BCL-2*, *MCL-1*, and *BIM1*. (**C**) RT-PCR of MLL-AF4 siRNA-treated samples (three biological replicates) used for the nascent RNA-seq experiments. *Error bars* are SD. (**D**) Gene transcription as measured by nascent RNA-seq and presented as FPKM values in control-treated (*black bars)* and MLL-AF4 siRNA-treated (*gray bars*) SEM cells. Results are the average of three biological replicates, ***p (FDR) < 0.001. (**E**) The same data as in (**D**) normalized to the control samples to illustrate the extent of the gene transcription change. (**F**) RT-PCR in control-treated (*black bars*) and MLL-AF4 siRNA (*gray bars*)-treated SEM cells. Average of 13 biological replicates; error bars are SD. ***p < 0.001, *p < 0.05 using a Mann–Whitney *U* test. (**G**) Sample Western blots in two independent experiments in control-treated (−) and MLL-AF4 siRNA-treated (+) SEM cells using the antibodies indicated.

A survey of MLL-AF4 ChIP-seq data 17, 18, 25, 26 revealed that only *BCL-2* and *MCL-1* were bound by MLL-AF4 in three data sets (although binding at *BIM* and *NOXA* was detected in at least two of these) (Fig. 1A). Because BIM protein levels are significantly increased in MLL-AF4 leukemias [18], we wanted to determine if lack of MLL-AF4 binding to *BIM* in the Wilkinson/Geng data set 17, 26 (an RS4;11 MLL-AF4 ALL cell line) was simply due to sensitivity of the ChIP-seq experiment. Using ChIP Q-PCR as a validation approach, we were able to detect a low level of MLL-AF4 signal at *BIM* in the RS4;11 cell line (Fig. 1B), thus indicating that in addition to *BCL-2* and *MCL-1*, *BIM* is a direct target of MLL-AF4 binding.

To explore MLL-AF4 regulation of BCL-2 family members further, we employed a highly sensitive assay for gene transcription by purifying nascent RNA transcripts coupled with next-generation sequencing (nascent RNA-seq). Although steady-state RNA levels are dependent on both RNA stability and transcription, nascent RNA production is reflective of transcription rate alone, allowing for a more sensitive measure of gene regulation. Using three independent MLL-AF4 siRNA knockdowns (Fig. 1C), we observed a reduction in transcription of the canonical MLL-AF4 target *RUNX1* and statistically significant but smaller reductions in *BCL-2* and *MCL-1* transcription, but not *BCL-XL* or *BCL-W* (Fig. 1D, E).

Using RT-PCR, we previously found that *MCL-1* RNA levels were unaffected by MLL-AF4 knockdowns [18]. To increase the robustness of this result, we performed Q-RT-PCR for *MCL-1* and other factors in up to 13 independent MLL-AF4 siRNA replicates using several independent primer sets (Fig. 1F). Similar to what we observed previously, MLL-AF4 knockdowns have a significant effect on *RUNX1* and *BCL-2* steady-state RNA levels, but no change in steady-state RNA was observed for *BCL-XL*. Four independent *MCL-1* primer sets exhibited a trend of reduced steady-state RNA levels, but the downregulation was not as significant as that observed with *BCL-2* (Fig. 1F).

MLL-AF4 siRNA treatment also increases PARP1 cleavage (a mark of apoptosis) and reduces both RUNX1 and BCL-2 protein levels (Fig. 1G), but surprisingly also reduces BCL-XL and has a strong effect on MCL-1 protein levels (Fig. 1G) despite the relatively minor effect on MCL-1 steady-state RNA (Fig. 1F). MCL-1 is also significantly regulated at the protein level [45], and βTrcP is an F-box–containing protein that can target MCL-1 for degradation [46]. βTrcP is not upregulated in MLL-AF4 knockdowns (Fig. 1G), but it remains possible that MLL-AF4 siRNA treatment somehow stimulates MCL-1 and perhaps BCL-XL protein degradation pathways in other ways. Combined with the ChIP-seq data, these results indicate that only *BCL-2* and *MCL-1* are direct transcriptional targets of MLL-AF4-mediated activation; however, MLL-AF4 may also possibly affect protein degradation pathways in the cell.

### MLL-AF4 causes increased *BIM* repression through recruitment of the PRC1 component CBX8

**Figure 2:**
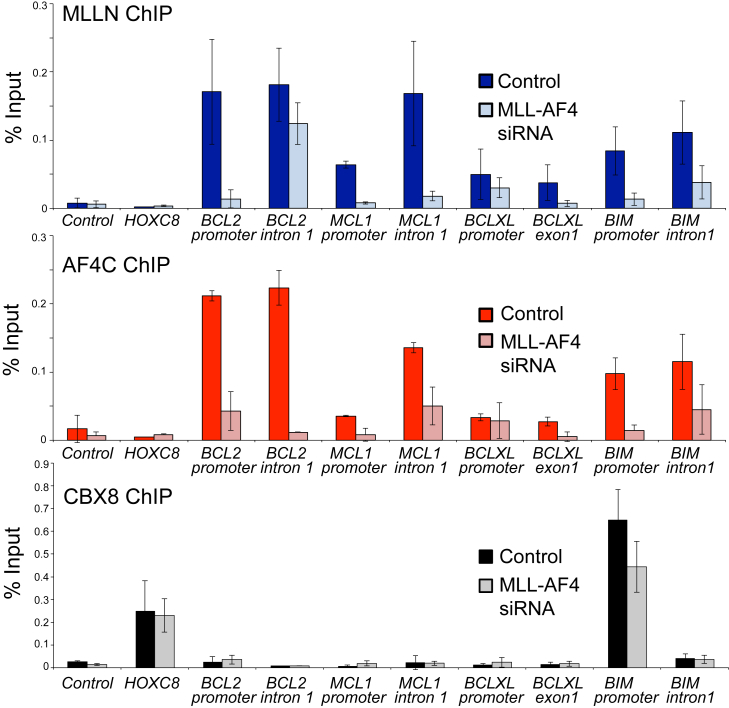
Loss of MLL-AF4 binding causes reduced binding of the PRC1 protein CBX8 to *BIM*. ChIP in MLL-AF4 siRNA (*light color*) or control-treated (*dark color*) SEM cells using MLL(N), AF4(C), and CBX8 antibodies at *BCL-2*, *MCL-1*, *BCL-XL*, and *BIM1*. *Bars* represent averages of three biological replicates (the same samples as those used to generate the nascent RNA-seq data in Fig. 1F) and error bars = SD.

Interestingly, loss of MLL-AF4 was associated with an increase in transcription of the proapoptotic *BIM* gene (Fig. 1D–F), although BIM protein levels are not obviously increased (Fig. 1G). MLL-FP activity at some gene targets has been found to be associated with the PRC1 protein CBX8 47, 48, a protein most commonly associated with gene repression. Strikingly, CBX8 displays robust binding to the *BIM* promoter, but not to any other of the *BCL-2* family genes studied (Fig. 2, bottom). Loss of MLL-AF4 binding at *BIM* was also associated with a loss of CBX8 binding (Fig. 2), whereas there was no change in CBX8 binding at a non-MLL-AF4-repressed gene target such as *HOXC8* (Fig. 2). This suggests that MLL-AF4 may cause repression of *BIM* transcription through recruitment of CBX8.

### DOT1L activity mirrors MLL-AF4 activity at *BCL-2* and *MCL-1*

**Figure 3:**
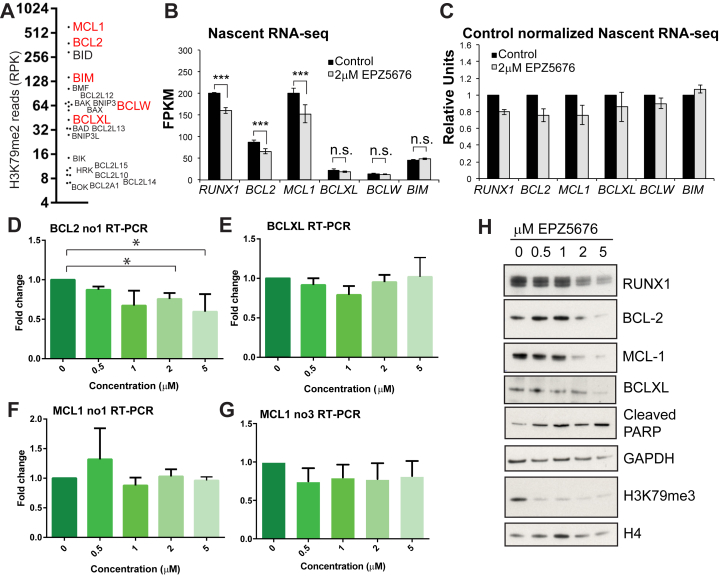
Treatment with the DOT1L inhibitor EPZ5676 and loss of H3K79me2/3 causes reduced *BCL-2* and *MCL-1* transcription, but has no effect on *BIM*. (**A**) BCL-2 family genes ranked by levels of H3K79me2 with *MCL-1*, *BCL-2*, *BID*, and *BIM* displaying the top four highest levels of H3K79me2. Read count represents number of H3K79me2 reads over the entire gene, normalized to the length of the gene (RPK). (**B**) Gene transcription as measured by nascent RNA-seq and presented as FPKM values in control-treated (*black bars*) versus 2μM EPZ5676-treated (*gray bars*) SEM cells. Results are averages of three biological replicates. ***p (FDR) < 0.001. (**C**) The same data as in (**B**) normalized to the control samples to illustrate the extent of the gene transcription change. (**D–G**) RT-PCR of *MCL-1*, *BCL-2*, and *BCL-XL* in SEM cells treated with different dosages of the DOT1L inhibitor EPZ5676. Average of six independent experiments except (**G**), which was three independent experiments. *p < 0.05 using the Mann–Whitney *U* test. (**H**) Sample Western blots of one set of samples from (**B**) for the proteins/marks indicated. GAPDH and H4 are shown as loading controls.

Our past work has suggested that MLL-AF4 stabilizes binding of DOT1L to gene targets and increases H3K79me2/3 17, 18. In the BCL-2 family, the *MCL-1* and *BCL-2* genes display the highest levels of H3K79me2 (Fig. 3A). Consistent with the MLL-AF4 siRNA data, treatment with the DOT1L inhibitor EPZ5676 results in reduced *BCL-2*, *MCL-1*, and *RUNX1* transcription (Fig. 3B,C), as well as a less robust effect on steady-state RNA levels (Fig. 3D–G). Interestingly, there is no effect on *BIM* expression following DOT1L inhibition, suggesting that upregulation of BIM is dependent on MLL-AF4-mediated CBX8 recruitment and is independent of H3K79me2 changes.

The relatively small changes in gene expression are contrasted with a strong reduction in RUNX1, BCL-2, and MCL-1 protein levels (Fig. 3H), similar to what was observed with MLL-AF4 siRNA treatment. This, combined with the observation that EPZ5676 treatment also causes an increase in PARP1 cleavage (Fig. 3H), suggests that MLL-AF4 and H3K79me2/3 both have a similar impact on cellular apoptotic pathways. Taken together, the data so far indicate that MLL-AF4 directly activates *MCL-1* and *BCL-2* through increased H3K79me2/3 levels.

### *BCL-2* has an enhancer at the 3′ end of the gene

**Figure 4:**
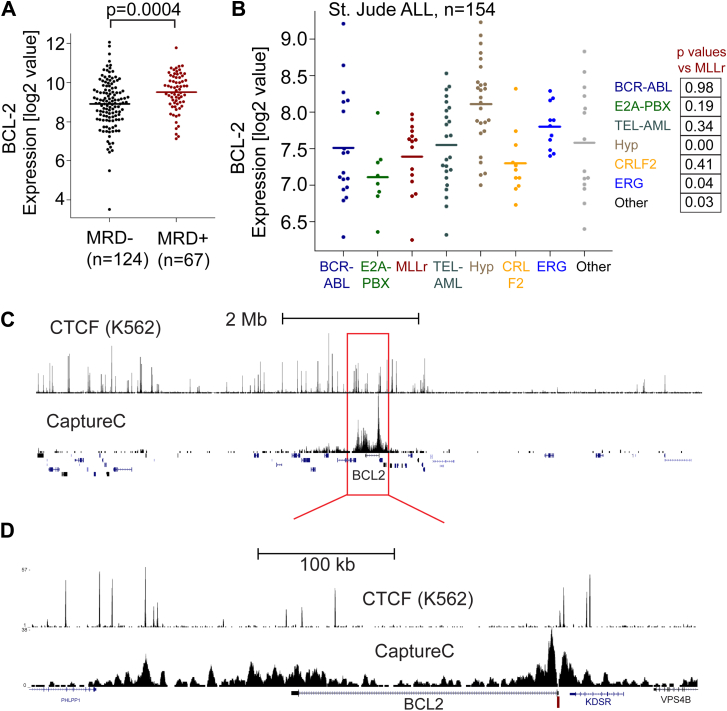
*BCL-2* is highly expressed in many ALL patient samples, and Capture-C identifies an enhancer cluster at the 3′ end of the *BCL-2* gene. (**A**) COG P9906 B-ALL patients (*n* = 207). Average expression of *BCL-2* is higher in MRD+ than MRD–patients. Each point represents an individual patient. (**B**) A St. Jude ALL data set [37] was assessed for *BCL-2* gene expression. Details of the data set are given under Methods. (**C**) CTCF ChIP-seq (top track, from K562 cells, encode track) compared with Capture-C (bottom track) using a capture probe designed to the BCL-2 promoter (*dark red bar*). The tracks indicate the region about 4 Mb on either side of *BCL-2*. (**D**) Blowup of *red box* region from (**C**), revealing that there is a major interaction cluster at the 3′ end of the *BCL-2* gene associated with several CTCF ChIP-seq peaks.

Patients with B-ALL with increased *BCL-2* gene expression at diagnosis have a significantly higher incidence of minimal residual disease (MRD+), a marker of relapse potential (Fig. 4A). Although *BCL-2* gene expression is upregulated in MLLr patients compared with normal pre-B cells [18], MLLr patients do not stand out as having significantly higher levels of *BCL-2* expression compared with other patient subtypes [18] (Fig. 4B). This indicates *BCL-2* overexpression is relatively common in leukemia, highlighting the importance of better understanding all aspects of *BCL-2* gene regulation.

Using a recently published chromosome conformation capture technique (Next-Generation Capture-C) that provides high-resolution enhancer–promoter interaction maps at single-locus resolution [39], we identified a tight cluster of interactions in SEM cells between the *BCL-2* promoter and other nearby sites (Fig. 4C, D). On comparison of our Capture-C data with CTCF ChIP-seq data (an ENCODE data set from K562 cells, ENCSR000DMA) that demarcates compartment boundaries, the major interaction site appears to be clustered at the 3′ end of *BCL-2* (Fig. 4C, D), thus marking a putative 3′ enhancer in MLL-AF4 leukemia cells.

**Figure 5:**
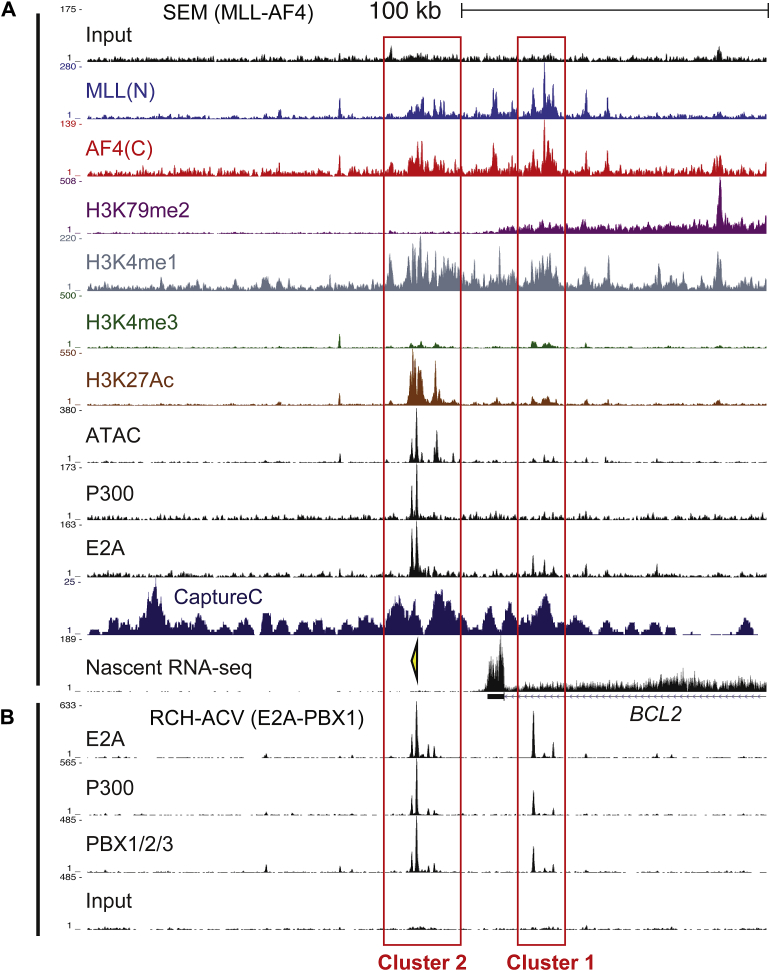
There are two main clusters of the *BCL-2* enhancer marked by H3K27Ac and bound by P300 in MLL-AF4 and E2A-PBX1 cells. (**A**) ChIP-seq data as indicated in SEM cells. *Red boxes* highlight the two enhancer ATAC/H3K27Ac clusters. The *yellow arrowhead* indicates a PCR primer set used for ChIP Q-PCR *BCL-2* enhancer experiments in Figure 6. (**B**) ChIP-seq in RCH-ACV (E2A-PBX1) cells reveals binding of E2A-PBX1 and P300 at the two enhancer clusters.

Further supporting this observation, we observed two main clusters in the putative 3′ enhancer (*red boxes*, Fig. 5) that are marked by H3K27 acetylation (H3K27Ac, a mark commonly associated with enhancer activation), are regions of open chromatin (as demarcated by ATAC-seq signal), and are enriched for H3K4me1 rather than H3K4me3 (a common signature of enhancers). The enhancer is also bound by MLL-AF4, P300, and wild-type E2A and is marked by P300 in an E2A-PBX1 leukemia line (Fig. 5A, B).

### MLL-AF4 binding controls H3K27Ac levels at the *BCL-2* enhancer

**Figure 6:**
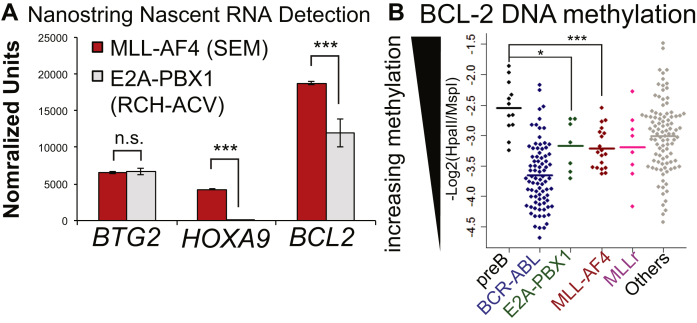

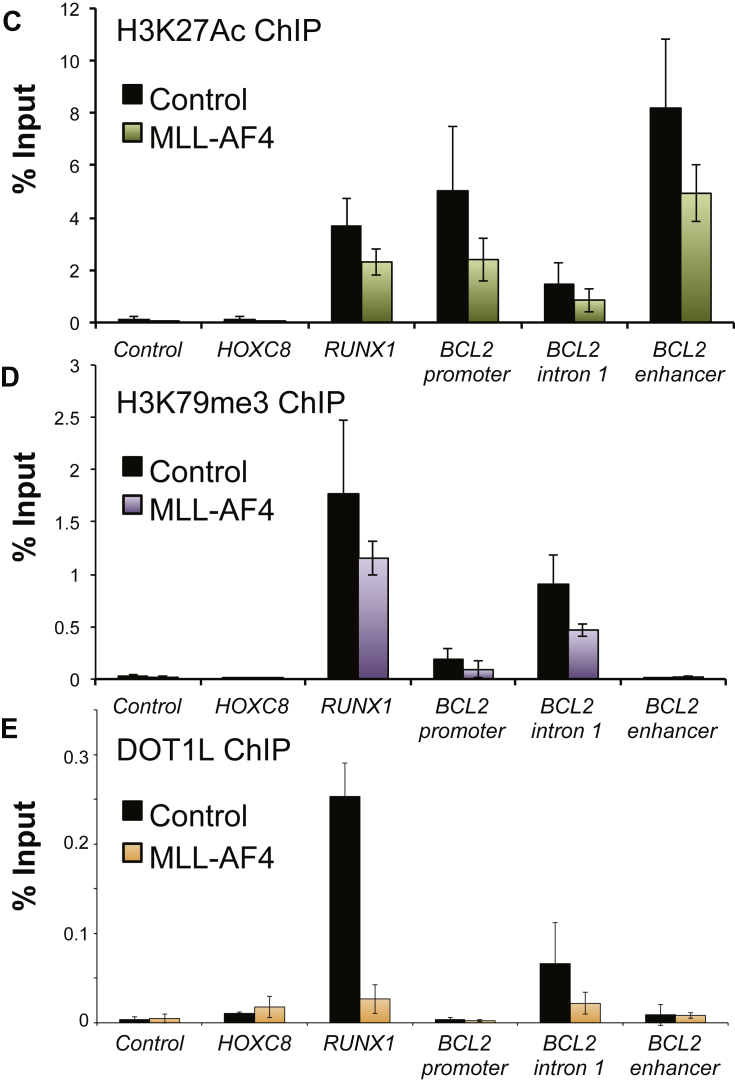
MLL-AF4 controls both H3K27Ac and H3K79me3. (**A**) Nascent RNA purified from SEM (MLL-AF4, *red bars*) and RCH-ACV (E2A-PBX1, *gray bars*) and quantified on a NanoString probe array. Average of two replicates. *BTG2* is a gene target transcribed at equal levels in both samples and is presented as a sample control. *HOXA9* (a canonical MLL-AF4 target gene) and *BCL-2* both display reduced transcription in the E2A-PBX1 sample. ***p < 0.001, NanoStriDE sequencing normalized. (**B**) BCL2 methylation data from the ECOG E2993 ALL trial (*n* = 215) revealing reduced DNA methylation at *BCL-2* in E2A-PBX1 (*green*) and MLL-AF4 (*red*) patients. Each point represents one patient or normal sample; the bar is the average value. ***p < 0.001, *p < 0.05. (**C–E**) ChIP in MLL-AF4 siRNA treated SEM cells (*colored*) compared with control (*black*) using antibodies to H3K27Ac, H3K79me3, or DOT1L. Bars represent the averages of three independent experiments; *error bars* are SD.

Unlike other patient samples, leukemias containing the E2A-PBX1 fusion protein consistently exhibit *BCL-2* expression levels similar to those of normal pre-B cells and lower than those of MLL-r and other leukemias [18] (Fig. 6A). Because *BCL-2* expression in E2A-PBX1 leukemias is comparable to that in normal pre-B cells, we reasoned that this leukemia subtype may be a useful point of comparison with MLL-AF4 cells.

E2A-PBX1 functions by binding mainly to enhancers and intergenic regions [26] and recruiting the acetyltransferase CBP/P300, causing inappropriate activation of target genes [49]. E2A-PBX1 and MLL-AF4 patient samples are associated with DNA hypomethylation, both genomewide and at *BCL-2*, compared with normal pre-B cells [26] (Fig. 6B), and both MLL-AF4 and E2A-PBX1 are bound to the *BCL-2* enhancer along with P300 (Fig. 5A, B). Interestingly, in MLL-AF4 siRNA knockdown cells (the same cells as in Fig. 1F, G), we observed reduced H3K27Ac (Fig. 6C), as well as reduced H3K79me3 and DOT1L binding (Fig. 6D, E). Thus, MLL-AF4 may promote *BCL-2* gene expression by binding to the gene body and the enhancer and through a combined effect on both H3K79me3 and H3K27Ac levels.

## Discussion

Cells are dependent on BCL-2, BCL-XL, and/or MCL-1 for their survival in a cell type–specific manner 50, 51. ABT-199 (venetoclax) is a new BH3 mimetic that was developed to specifically target BCL-2 while sparing BCL-XL, hence avoiding thrombocytopenia 27, 52, 53, 54, 55. Venetoclax has been found to have high antileukemia activity in patients with chronic lymphocytic leukemia (CLL) [56] and has been reported to have preclinical activities in estrogen receptor–positive breast cancer, acute myeloid leukemia, early T-cell progenitor leukemia, and Myc-driven B-cell lymphomas 27, 52, 53, 54, 55. However, venetoclax is less effective than navitoclax (which targets both BCL-2 and BCL-XL) as a monotherapy in most ALL samples [28], with the notable exception of MLLr ALL leukemias 18, 28. The reasons that MLLr samples are particularly addicted to BCL-2 are likely complex, but this current work suggests that there may be additional regulatory interactions on the protein level in MLL-AF4 leukemias.

Even in MLLr leukemias, however, some patient samples are resistant to venetoclax treatment alone [28] or they relapse after an initial response [18]. Acquisition of venetoclax resistance is often associated with increased MCL-1 and BCL-XL protein levels and activation of the AKT pathway [33]. We previously found that venetoclax synergizes with DOT1L inhibitors [18], and one explanation for this synergy could be that DOT1L inhibition affects both BCL-2 and MCL-1 protein levels (Fig. 3H). Considering that the changes in transcription of *MCL-1* and *BCL-2* are relatively moderate on treatment with DOT1L inhibitors, it is however surprising that there is such a strong effect on both MCL-1 and BCL-2 at the protein level (Fig. 3H).

Of all the BCL-2 family members, only *BCL-2* and *MCL-1* are directly activated by MLL-AF4; *BIM* appears to be partially repressed by MLL-AF4 through recruitment of CBX8. MLL-AF4 can increase ENL recruitment 17, 18, and ENL has been found to directly interact with CBX8 at gene targets 48, 57, so it is possible that MLL-AF4 recruits CBX8 to *BIM* via ENL. However, because ENL can inhibit CBX8-repressive activity [48], it is unclear if an MLL-AF4/ENL complex can recruit CBX8 to *BIM* and cause repression. Further work is necessary to explore the interesting possibility of whether MLL-AF4 can act as a repressor for a subset of gene targets. Nevertheless, this observation may not be functionally important in patients, as BIM protein levels are significantly higher in MLL-AF4 leukemias than in other ALL samples [18].

From our results, *BCL-2* appears to be the major target of MLL-AF4 regulation in the BCL-2 apoptotic pathway. Because of this, we further investigated MLL-AF4 regulation of *BCL-2* and identified a potential enhancer that contains two clusters of H3K27Ac. H3K27Ac is deposited by the acetyltransferase P300 and it is thought to stabilize binding of chromatin complexes to promoters and enhancers to promote gene activation [58]. In MLL-AF4 cells, P300 is bound to the *BCL-2* enhancer, and loss of MLL-AF4 results in loss of both H3K79me3 in the gene body and H3K27Ac at the enhancer. It has recently been observed that on treatment with DOT1L inhibitors, H3K79 methylation loss is followed by a loss of P300 binding at gene targets [59]. Along with our data here, this suggests a link between MLL-AF4 activity, H3K79Me2/3 in the gene body, and P300 activity at enhancers. Further investigation is necessary to understand the exact connections between these factors and enhancer activity. However, our data suggest that MLL-AF4 promotes both H3K79me3 and H3K27Ac to stimulate increased expression of *BCL-2*.
